# The genome sequence of the Figure of Eight, *Diloba caeruleocephala *(Linnaeus, 1758)

**DOI:** 10.12688/wellcomeopenres.20497.1

**Published:** 2023-12-05

**Authors:** Liam M. Crowley, Finley Hutchinson, University of Oxford and Wytham Woods Genome Acquisition Lab, Darwin Tree of Life Barcoding collective, Wellcome Sanger Institute Tree of Life Management, Samples and Laboratory team, Wellcome Sanger Institute Scientific Operations: Sequencing Operations, Wellcome Sanger Institute Tree of Life Core Informatics team, Tree of Life Core Informatics collective, Darwin Tree of Life Consortium

**Affiliations:** 1 University of Oxford, Oxford, England, UK; 2 University of Exeter, Penryn, England, UK

**Keywords:** Diloba caeruleocephala, Figure of Eight, genome sequence, chromosomal, Lepidoptera

## Abstract

We present a genome assembly from an individual male *Diloba caeruleocephala* (the Figure of Eight; Arthropoda; Insecta; Lepidoptera; Noctuidae). The genome sequence is 762.5 megabases in span. Most of the assembly is scaffolded into 31 chromosomal pseudomolecules, including the Z sex chromosome. The mitochondrial genome has also been assembled and is 15.36 kilobases in length.

## Species taxonomy

Eukaryota; Metazoa; Eumetazoa; Bilateria; Protostomia; Ecdysozoa; Panarthropoda; Arthropoda; Mandibulata; Pancrustacea; Hexapoda; Insecta; Dicondylia; Pterygota; Neoptera; Endopterygota; Amphiesmenoptera; Lepidoptera; Glossata; Neolepidoptera; Heteroneura; Ditrysia; Obtectomera; Noctuoidea; Noctuidae; Dilobinae; *Diloba*; *Diloba caeruleocephala* (Linnaeus, 1758) (NCBI:txid987926).

## Background

The Figure of Eight *Diloba caeruleocephala* (Linnaeus, 1758) is an endangered moth in Britain, having suffered a 96% decrease in abundance between 1970 and 2016 as well as a decline in its distribution ( Randle *et al.*, 2019). Within the family Noctuidae, it is the sole British representative of the subfamily Dilobinae, and is a distinctive species named for the white markings on the forewing, the inner of which resembles an ‘8’. The most likely confusion species in the British Isles are probably the Lasiocampids *Tethea ocularis* (Linnaeus, 1767), the Figure of Eighty, and *Tethea or* ([Denis & Schiffermüller], 1775), the Poplar Lutestring, which have similar white markings but otherwise very different forewing patterning and lack serrated antennae in the male.

*D. caeruleocephala* overwinters as an egg, with the larvae hatching in late April. Larvae feed on the leaves of a large variety of trees and shrubs (particularly *Prunus spinosa* and *Crataegus* spp.), before pupating on or under the ground in a silken cocoon. The larvae are pale grey with large black pinacula, yellow dorsal and lateral spots and a raised yellow band across the second abdominal segment ( Henwood *et al.*, 2020). The life cycle of the species was studied in detail by Bolu & Özgen (2007).

Despite the rapid decline of the species in Britain, in Ireland (where it has a very scattered distribution) it has fared better, seeing a significant increase in records from just five before 1970 to twenty-six between 2000 and 2019 ( Randle *et al.*, 2019). A synthetic sex-attractant for the species was presented by Subchev *et al.* (2000).

We present a chromosomally complete genome sequence for *Diloba caeruleocephala*, based on one male specimen from Wytham Woods, Oxfordshire, as part of the Darwin Tree of Life Project. This project is a collaborative effort to sequence all named eukaryotic species in the Atlantic Archipelago of Britain and Ireland.

## Genome sequence report

**Figure 1.**
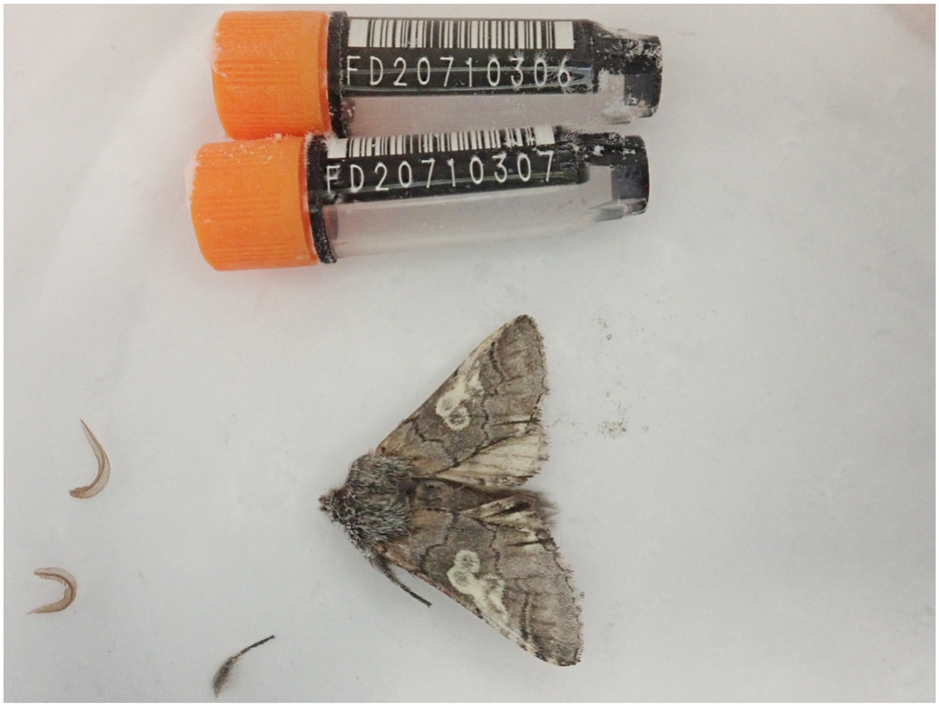
Photograph of the *Diloba caeruleocephala* (ilDilCaer1) specimen used for genome sequencing.

The genome was sequenced from one male *Diloba caeruleocephala* ( Figure 1) collected from Wytham Woods, Oxfordshire, UK (51.77, –1.34). A total of 30-fold coverage in Pacific Biosciences single-molecule HiFi long reads was generated. Primary assembly contigs were scaffolded with chromosome conformation Hi-C data. Manual assembly curation corrected 2 missing joins or mis-joins and removed one haplotypic duplication.

**Figure 2.**
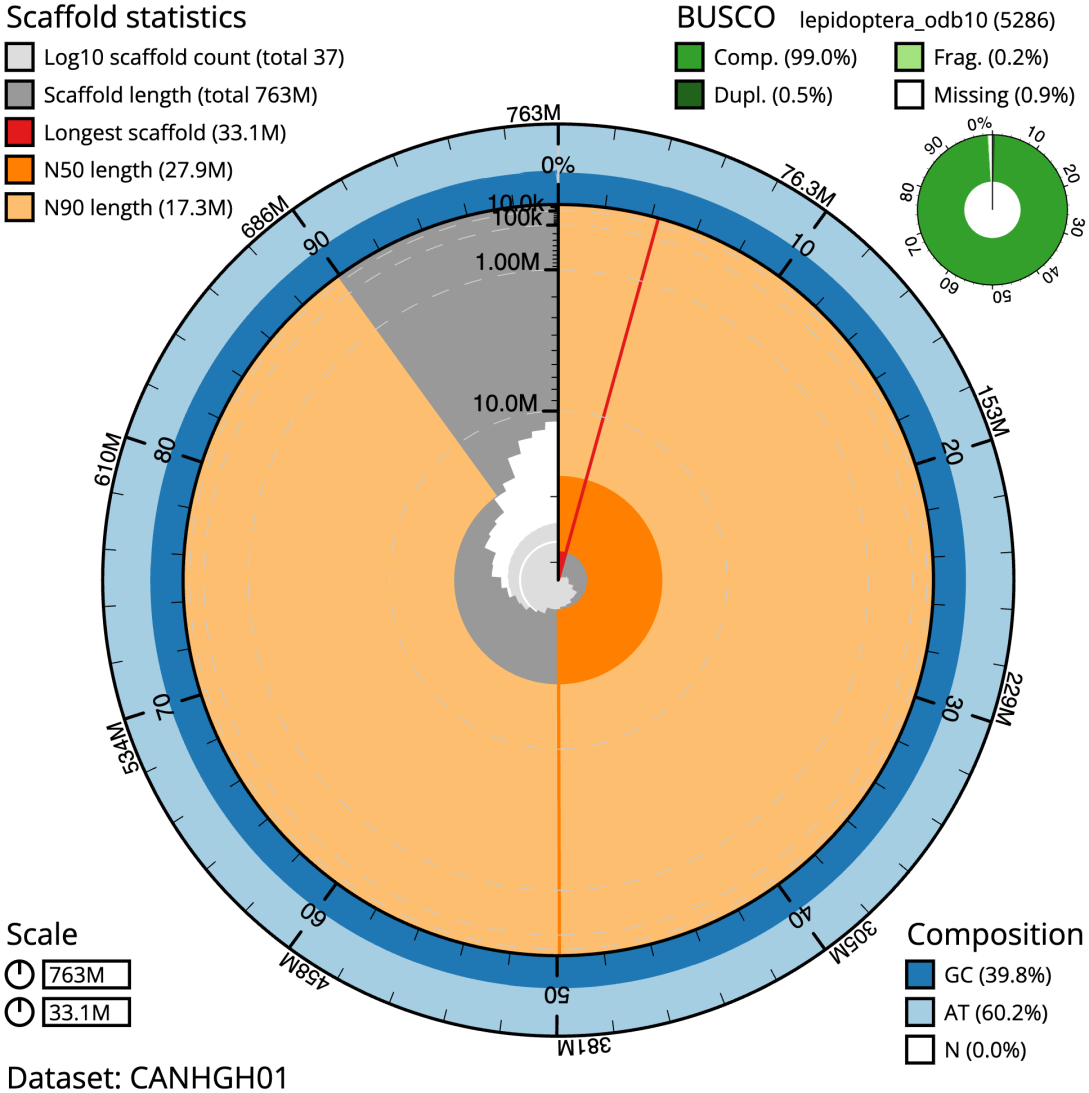
Genome assembly of *Diloba caeruleocephala*, ilDilCaer1.1: metrics. The BlobToolKit Snailplot shows N50 metrics and BUSCO gene completeness. The main plot is divided into 1,000 size-ordered bins around the circumference with each bin representing 0.1% of the 762,531,772 bp assembly. The distribution of scaffold lengths is shown in dark grey with the plot radius scaled to the longest scaffold present in the assembly (33,052,791 bp, shown in red). Orange and pale-orange arcs show the N50 and N90 scaffold lengths (27,899,106 and 17,255,644 bp), respectively. The pale grey spiral shows the cumulative scaffold count on a log scale with white scale lines showing successive orders of magnitude. The blue and pale-blue area around the outside of the plot shows the distribution of GC, AT and N percentages in the same bins as the inner plot. A summary of complete, fragmented, duplicated and missing BUSCO genes in the lepidoptera_odb10 set is shown in the top right. An interactive version of this figure is available at https://blobtoolkit.genomehubs.org/view/Diloba%20caeruleocephala/dataset/CANHGH01/snail.

**Figure 3.**
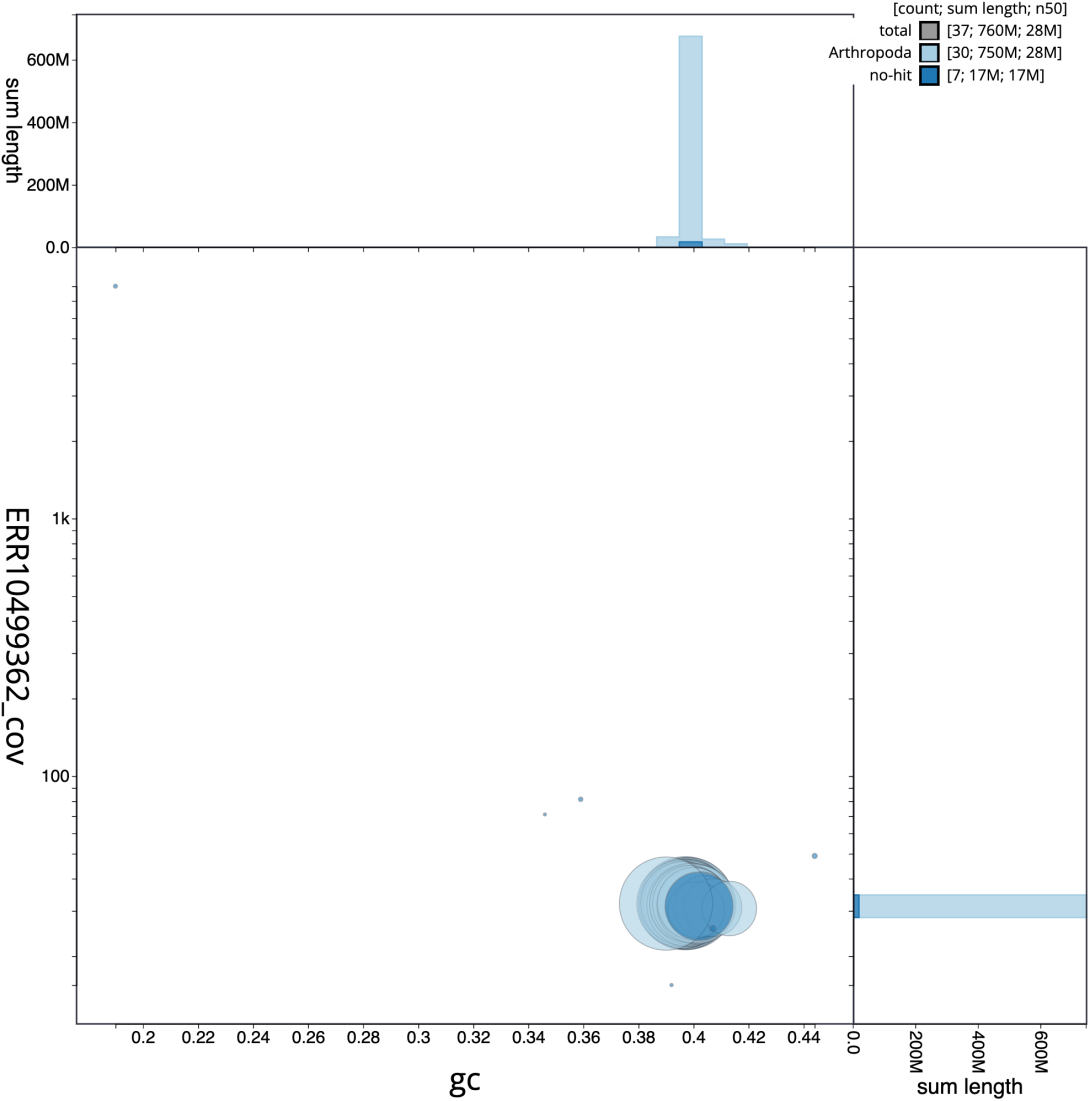
Genome assembly of *Diloba caeruleocephala*, ilDilCaer1.1: BlobToolKit GC-coverage plot. Scaffolds are coloured by phylum. Circles are sized in proportion to scaffold length. Histograms show the distribution of scaffold length sum along each axis. An interactive version of this figure is available at https://blobtoolkit.genomehubs.org/view/Diloba%20caeruleocephala/dataset/CANHGH01/blob.

**Figure 4.**
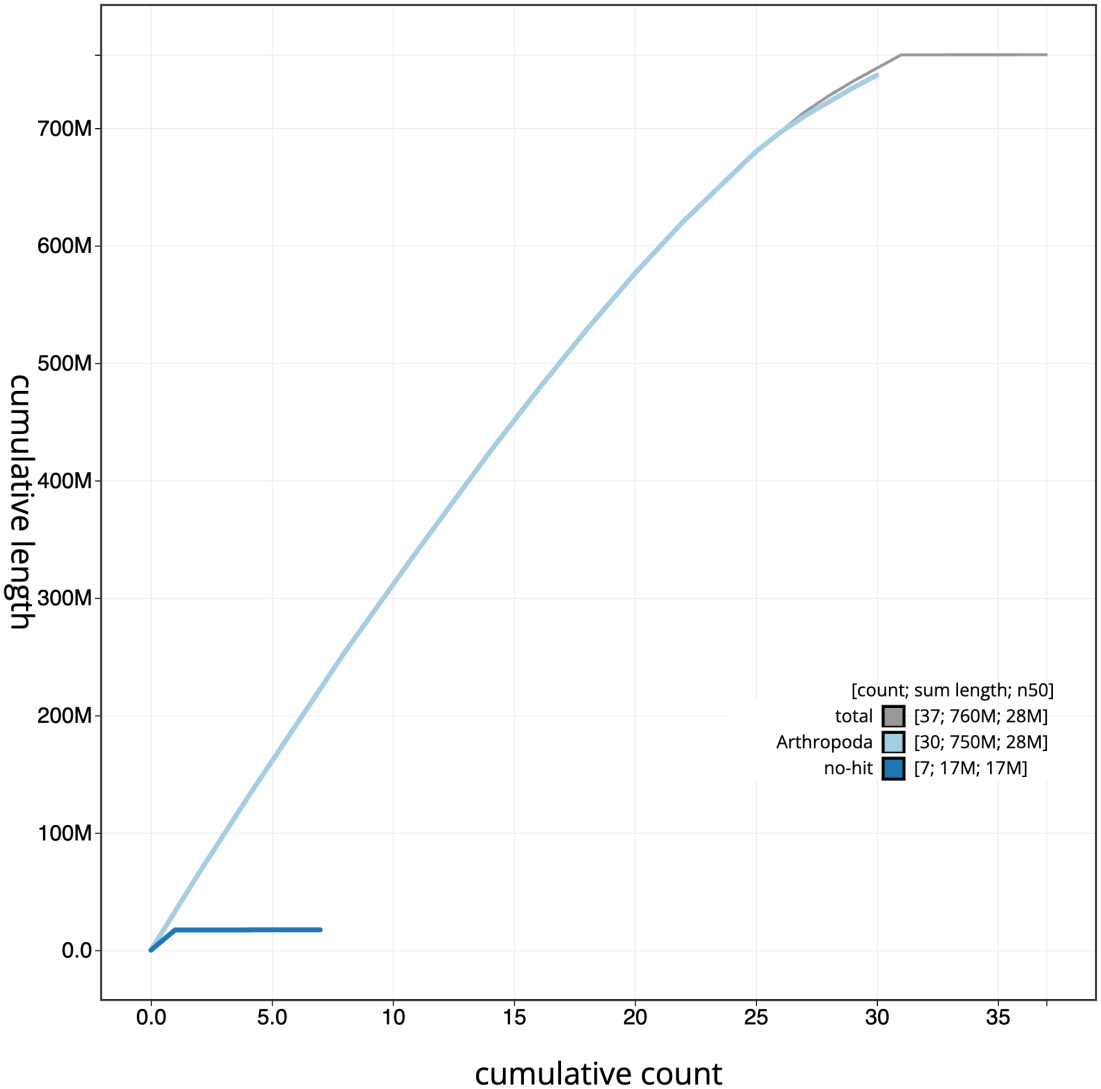
Genome assembly of *Diloba caeruleocephala*, ilDilCaer1.1: BlobToolKit cumulative sequence plot. The grey line shows cumulative length for all scaffolds. Coloured lines show cumulative lengths of scaffolds assigned to each phylum using the buscogenes taxrule. An interactive version of this figure is available at https://blobtoolkit.genomehubs.org/view/Diloba%20caeruleocephala/dataset/CANHGH01/cumulative.

**Figure 5.**
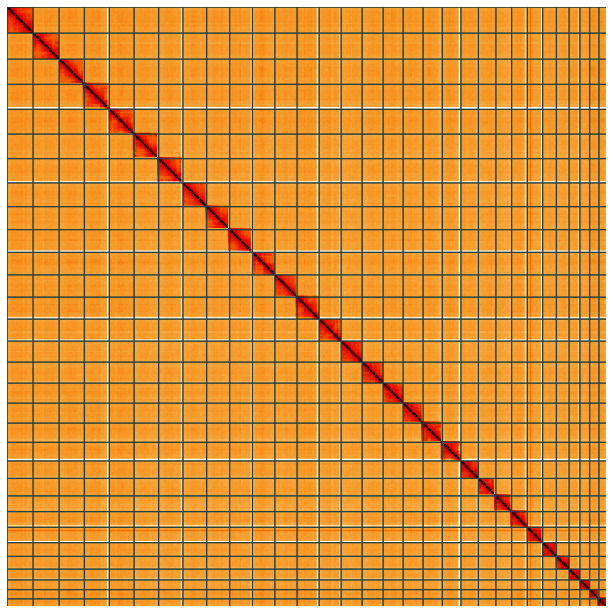
Genome assembly of *Diloba caeruleocephala*, ilDilCaer1.1: Hi-C contact map of the ilDilCaer1.1 assembly, visualised using HiGlass. Chromosomes are shown in order of size from left to right and top to bottom. An interactive version of this figure may be viewed at https://genome-note-higlass.tol.sanger.ac.uk/l/?d=RGHSoMHwRhCT5dxmF8w16w.

The final assembly has a total length of 762.5 Mb in 36 sequence scaffolds with a scaffold N50 of 27.9 Mb ( Table 1). The snailplot in Figure 2 provides a summary of the assembly statistics, while the distribution of reads on GC proportion and coverage is shown in Figure 3. The cumulative assembly plot in Figure 4 shows curves for subsets of scaffolds assigned to different phyla. Most (99.98%) of the assembly sequence was assigned to 31 chromosomal-level scaffolds, representing 30 autosomes and the X sex chromosome. Chromosome-scale scaffolds confirmed by the Hi-C data are named in order of size ( Figure 5; Table 2). While not fully phased, the assembly deposited is of one haplotype. Contigs corresponding to the second haplotype have also been deposited. The mitochondrial genome was also assembled and can be found as a contig within the multifasta file of the genome submission.

**Table 1.** Genome data for *Diloba caeruleocephala*, ilDilCaer1.1.

Project accession data		
Assembly identifier	ilDilCaer1.1	
Species	*Diloba caeruleocephala*	
Specimen	ilDilCaer1	
NCBI taxonomy ID	987926	
BioProject	PRJEB57680	
BioSample ID	SAMEA110451510	
Isolate information	ilDilCaer1, male: head and thorax (DNA sequencing, Hi-C data, RNA sequencing)	
Assembly metrics *		*Benchmark*
Consensus quality (QV)	65.6	*≥ 50*
*k*-mer completeness	100%	*≥ 95%*
BUSCO **	C:99.0%[S:98.4%,D:0.5%],F:0.2%,M:0.9%,n:5,286	*C ≥ 95%*
Percentage of assembly mapped to chromosomes	99.98%	*≥ 95%*
Sex chromosomes	Z chromosome	*localised homologous pairs*
Organelles	Mitochondrial genome assembled	*complete single alleles*
Raw data accessions		
PacificBiosciences SEQUEL II	ERR10499362	
Hi-C Illumina	ERR10501028	
PolyA RNA-Seq Illumina	ERR11606296	
Genome assembly		
Assembly accession	GCA_947459985.1	
*Accession of alternate haplotype*	GCA_947459995.1	
Span (Mb)	762.5	
Number of contigs	137	
Contig N50 length (Mb)	9.8	
Number of scaffolds	36	
Scaffold N50 length (Mb)	27.9	
Longest scaffold (Mb)	33.1	

* Assembly metric benchmarks are adapted from column VGP-2020 of “Table 1: Proposed standards and metrics for defining genome assembly quality” from ( Rhie *et al.*, 2021). ** BUSCO scores based on the lepidoptera_odb10 BUSCO set using v5.3.2. C = complete [S = single copy, D = duplicated], F = fragmented, M = missing, n = number of orthologues in comparison. A full set of BUSCO scores is available at https://blobtoolkit.genomehubs.org/view/Diloba%20caeruleocephala/dataset/CANHGH01/busco.

**Table 2.** Chromosomal pseudomolecules in the genome assembly of *Diloba caeruleocephala*, ilDilCaer1.

INSDC accession	Chromosome	Length (Mb)	GC%
OX381605.1	1	33.04	39.5
OX381606.1	2	32.16	40.0
OX381607.1	3	31.77	39.5
OX381608.1	4	31.24	40.0
OX381609.1	5	31.08	39.5
OX381610.1	6	31.01	39.5
OX381611.1	7	30.11	39.5
OX381612.1	8	29.07	39.5
OX381613.1	9	28.99	39.5
OX381614.1	10	28.49	40.0
OX381615.1	11	28.32	39.5
OX381616.1	12	27.9	40.0
OX381617.1	13	27.89	39.5
OX381618.1	14	26.83	39.5
OX381619.1	15	26.41	39.5
OX381620.1	16	25.62	40.0
OX381621.1	17	25.35	40.0
OX381622.1	18	24.39	40.0
OX381623.1	19	23.59	40.0
OX381624.1	20	22.19	40.0
OX381625.1	21	22.0	40.0
OX381626.1	22	20.24	40.0
OX381627.1	23	19.87	40.0
OX381628.1	24	19.4	40.0
OX381629.1	25	17.26	40.0
OX381630.1	26	16.27	40.0
OX381631.1	27	13.81	40.5
OX381632.1	28	12.37	40.5
OX381633.1	29	11.72	40.0
OX381634.1	30	10.93	41.5
OX381604.1	Z	33.05	39.0
OX381635.1	MT	0.02	19.0

The estimated Quality Value (QV) of the final assembly is 65.6 with *k*-mer completeness of 100%, and the assembly has a BUSCO v5.3.2 completeness of 99.0% (single = 98.4%, duplicated = 0.5%), using the lepidoptera_odb10 reference set ( *n* = 5,286).

Metadata for specimens, barcode results, spectra estimates, sequencing runs, contaminants and pre-curation assembly statistics are given at https://links.tol.sanger.ac.uk/species/987926.

## Methods

### Sample acquisition and nucleic acid extraction

A male *Diloba caeruleocephala* (specimen ID Ox001971, ToLID ilDilCaer1) was collected from Wytham Woods, Oxfordshire (biological vice-county Berkshire), UK (latitude 51.77, longitude –1.34) on 2021-10-10 using a light trap. The specimen was collected and identified by Liam Crowley (University of Oxford) and preserved on dry ice.

The workflow for high molecular weight (HMW) DNA extraction at the Wellcome Sanger Institute (WSI) includes a sequence of core procedures: sample preparation; sample homogenisation; DNA extraction; HMW DNA fragmentation; and fragmented DNA clean-up. The sample was prepared for DNA extraction at the WSI Tree of Life laboratory: the ilDilCaer1 sample was weighed and dissected on dry ice with tissue set aside for Hi-C sequencing ( https://dx.doi.org/10.17504/protocols.io.x54v9prmqg3e/v1). Tissue from the head and thorax was disrupted using a Nippi Powermasher fitted with a BioMasher pestle ( https://dx.doi.org/10.17504/protocols.io.5qpvo3r19v4o/v1). DNA was extracted at the WSI Scientific Operations core using the Qiagen MagAttract HMW DNA kit, according to the manufacturer’s instructions.

RNA was extracted from head and thorax tissue of ilDilCaer1 using the Automated MagMax™ *mir*Vana protocol ( https://dx.doi.org/10.17504/protocols.io.6qpvr36n3vmk/v1). The RNA concentration was assessed using a Nanodrop spectrophotometer and Qubit Fluorometer using the Qubit RNA Broad-Range (BR) Assay kit. Analysis of the integrity of the RNA was done using the Agilent RNA 6000 Pico Kit and Eukaryotic Total RNA assay.

Protocols developed by the Tree of Life laboratory are publicly available on protocols.io: https://dx.doi.org/10.17504/protocols.io.8epv5xxy6g1b/v1.

### Sequencing

Pacific Biosciences HiFi circular consensus and 10X Genomics read cloud DNA sequencing libraries were constructed according to the manufacturers’ instructions. Poly(A) RNA-Seq libraries were constructed using the NEB Ultra II RNA Library Prep kit. DNA and RNA sequencing was performed by the Scientific Operations core at the WSI on Pacific Biosciences SEQUEL II (HiFi) and Illumina NovaSeq 6000 (RNA-Seq) instruments. Hi-C data were also generated from remaining head and thorax tissue of ilDilCaer1 using the Arima2 kit and sequenced on the Illumina NovaSeq 6000 instrument.

### Genome assembly, curation and evaluation

Assembly was carried out with Hifiasm ( Cheng *et al.*, 2021) and haplotypic duplication was identified and removed with purge_dups ( Guan *et al.*, 2020). The assembly was then scaffolded with Hi-C data ( Rao *et al.*, 2014) using YaHS ( Zhou *et al.*, 2023). The assembly was checked for contamination and corrected as described previously ( Howe *et al.*, 2021). Manual curation was performed using HiGlass ( Kerpedjiev *et al.*, 2018) and Pretext ( Harry, 2022). The mitochondrial genome was assembled using MitoHiFi ( Uliano-Silva *et al.*, 2023), which runs MitoFinder ( Allio *et al.*, 2020) or MITOS ( Bernt *et al.*, 2013) and uses these annotations to select the final mitochondrial contig and to ensure the general quality of the sequence.

A Hi-C map for the final assembly was produced using bwa-mem2 ( Vasimuddin *et al.*, 2019) in the Cooler file format ( Abdennur & Mirny, 2020). To assess the assembly metrics, the *k*-mer completeness and QV consensus quality values were calculated in Merqury ( Rhie *et al.*, 2020). This work was done using Nextflow ( Di Tommaso *et al.*, 2017) DSL2 pipelines “sanger-tol/readmapping” ( Surana *et al.*, 2023a) and “sanger-tol/genomenote” ( Surana *et al.*, 2023b). The genome was analysed within the BlobToolKit environment ( Challis *et al.*, 2020) and BUSCO scores ( Manni *et al.*, 2021; Simão *et al.*, 2015) were calculated.

Table 3 contains a list of relevant software tool versions and sources.

**Table 3.** Software tools: versions and sources.

Software tool	Version	Source
BlobToolKit	4.1.7	https://github.com/blobtoolkit/blobtoolkit
BUSCO	5.3.2	https://gitlab.com/ezlab/busco
Hifiasm	0.16.1-r375	https://github.com/chhylp123/hifiasm
HiGlass	1.11.6	https://github.com/higlass/higlass
Merqury	MerquryFK	https://github.com/thegenemyers/MERQURY.FK
MitoHiFi	2	https://github.com/marcelauliano/MitoHiFi
PretextView	0.2	https://github.com/wtsi-hpag/PretextView
purge_dups	1.2.3	https://github.com/dfguan/purge_dups
sanger-tol/genomenote	v1.0	https://github.com/sanger-tol/genomenote
sanger-tol/ readmapping	1.1.0	https://github.com/sanger-tol/readmapping/tree/1.1.0
YaHS	1.2a	https://github.com/c-zhou/yahs

### Wellcome Sanger Institute – Legal and Governance

The materials that have contributed to this genome note have been supplied by a Darwin Tree of Life Partner. The submission of materials by a Darwin Tree of Life Partner is subject to the **‘Darwin Tree of Life Project Sampling Code of Practice’**, which can be found in full on the Darwin Tree of Life website here. By agreeing with and signing up to the Sampling Code of Practice, the Darwin Tree of Life Partner agrees they will meet the legal and ethical requirements and standards set out within this document in respect of all samples acquired for, and supplied to, the Darwin Tree of Life Project.

Further, the Wellcome Sanger Institute employs a process whereby due diligence is carried out proportionate to the nature of the materials themselves, and the circumstances under which they have been/are to be collected and provided for use. The purpose of this is to address and mitigate any potential legal and/or ethical implications of receipt and use of the materials as part of the research project, and to ensure that in doing so we align with best practice wherever possible. The overarching areas of consideration are:

•   Ethical review of provenance and sourcing of the material

•   Legality of collection, transfer and use (national and international)

Each transfer of samples is further undertaken according to a Research Collaboration Agreement or Material Transfer Agreement entered into by the Darwin Tree of Life Partner, Genome Research Limited (operating as the Wellcome Sanger Institute), and in some circumstances other Darwin Tree of Life collaborators.

## Data Availability

European Nucleotide Archive: *Diloba caeruleocephala* (figure of eight). Accession number PRJEB57680; https://identifiers.org/ena.embl/PRJEB57680 ( Wellcome Sanger Institute, 2022). The genome sequence is released openly for reuse. The *Diloba caeruleocephala* genome sequencing initiative is part of the Darwin Tree of Life (DToL) project. All raw sequence data and the assembly have been deposited in INSDC databases. The genome will be annotated using available RNA-Seq data and presented through the Ensembl pipeline at the European Bioinformatics Institute. Raw data and assembly accession identifiers are reported in Table 1.

## Author information

Members of the University of Oxford and Wytham Woods Genome Acquisition Lab are listed here: https://doi.org/10.5281/zenodo.7125292.

Members of the Darwin Tree of Life Barcoding collective are listed here: https://doi.org/10.5281/zenodo.4893703.

Members of the Wellcome Sanger Institute Tree of Life Management, Samples and Laboratory team are listed here: https://doi.org/10.5281/zenodo.10066175.

Members of Wellcome Sanger Institute Scientific Operations: Sequencing Operations are listed here: https://doi.org/10.5281/zenodo.10043364.

Members of the Wellcome Sanger Institute Tree of Life Core Informatics team are listed here: https://doi.org/10.5281/zenodo.10066637.

Members of the Tree of Life Core Informatics collective are listed here: https://doi.org/10.5281/zenodo.5013541.

Members of the Darwin Tree of Life Consortium are listed here: https://doi.org/10.5281/zenodo.4783558.
