# Supplementary material for: The genome sequence of the Figure of Eight, Diloba caeruleocephala (Linnaeus, 1758)
Source: Wellcome Open Res. 2023 Dec 5;8:554. [Version 1] doi: 10.12688/wellcomeopenres.20497.1 (PMC11282394; doi:10.12688/wellcomeopenres.20497.1)
Supplement: Peer review history

# Reviewer report: Reviewer response for version 1

**DOI:** 10.21956/wellcomeopenres.22690.r79370

**Published:** 2024-07-26

**Authors:** Mahamadi Kientega

**Affiliations:** 1 Entomology, Sya Innovation Center, Bobo-Dioulasso, Hauts-Bassins, Burkina Faso

In short, this manuscript presents a complete view of the genomic organisation of *Diloba caeruleocephala*. The rationale and the methodology are very well presented. 

 However, I found the results part insufficient, I was expecting some stories about the evolutionary pattern of the Diloba caeruleocephala species and the genomic relationship between the other species, as the tree of life tries to reconstruct the evolutionary relationship between species. 

 I'm also hoping that the authors will explain the figure 5 a bit more in terms of chromosomal rearrangements and genomic structuring.

Are sufficient details of methods and materials provided to allow replication by others?

Yes

Is the rationale for creating the dataset(s) clearly described?

Yes

Are the datasets clearly presented in a useable and accessible format?

Yes

Are the protocols appropriate and is the work technically sound?

Yes

Reviewer Expertise:

Malaria vector bio-ecology and genomic

I confirm that I have read this submission and believe that I have an appropriate level of expertise to confirm that it is of an acceptable scientific standard.

# Reviewer report: Reviewer response for version 1

**DOI:** 10.21956/wellcomeopenres.22690.r74125

**Published:** 2024-05-26

**Authors:** Maria Sharakhova

**Affiliations:** 1 Virginia Polytechnic and State University,, Blacksburg, VA, USA

The manuscript of Liam M. Crowley and Finley Hutchinson “ The genome of the Figure Eight, Diloba caeruleocephala” represents a brief report of de novo sequenced genome of an endangered in Britain moth species. The genome was generated from a single male using a cutting-edge technology sequencing Pacific Biosciences (HiFi) and Hi-C scaffolding approach. The genome assembly is 762.32 Mb in size and consists of 31 chromosomal pseudomolecules including Z sex chromosome. Mitochondrial genome was also assembled. The genome was sequenced as part of the Darvin Tree of Life project with the purpose of obtaining sequencing data for all named eukaryotic species in Britain and Ireland.

 Major concerns:

 Although this manuscript represents a brief report of the de novo genome assembly, I think that all important information about the generating of the genome assembly has to be provided here.

 The missing information in the manuscript text include the following:

1. It would be great if the authors provide description of different steps in the assembly development.
2. Please, describe how different methods used here were incorporated into developing the genome assembly. What was the purpose of using each method?
3. The purpose of obtaining RNA-seq data is not explained in the manuscript at all. Please, provide this information.
4. Please, explain what does it mean the assembly “was not fully phased”. Provide, some specific details of “not fully”.
5. It is unclear if cytogenetic evaluation of a chromosome number in this species or related species were conducted previously and does this number matches the number of chromosomal pseudomolecules generated by this project.
6. Please, also provide information about the companies from which specific reagents and kits were obtained.

I would also suggest to improve the quality of the figures:

 Figure 1. The purpose of showing microcentrifuge tubes on the figure 1 is unclear.  If tubes are shown to provide some scale for the size of the moth specimen, this has to be explained in the figure legend. However, it is better to use a ruler with scale in centimeters here. I would suggest to reformat this figure, provide a scale in centimeters and also remove some empty space around the specimen.

 Figure 2. The numbers on the scale in the center of the figure (100K, 10.0K) overlap and have to be corrected. In the right top corner, where BUSCO score is shown, it is unclear where different shades of green are shown in the circular diagram. They are simply invisible on the diagram. I would suggest to connect different fractions on the diagram by lines with the squares with different shades of green. Also, the scale which is shown in the left bottom corner of the figure has to be explained in the figure legend.

 Figure 5. I would suggest to add a scale in Mb to indicate the sizes of the chromosomal scaffolds. Please, also indicate which chromosome is considered as Z here.

 Minor concerns.

1. The sex chromosome is indicated as Z chromosome in abstract but as X chromosome in the manuscript text. Please, correct this.
2. In Figure 5 legend authors wrote “Chromosomes are shown in order of size”. It is better to write “in order of decreasing in size”.

Are sufficient details of methods and materials provided to allow replication by others?

Partly

Is the rationale for creating the dataset(s) clearly described?

Yes

Are the datasets clearly presented in a useable and accessible format?

Yes

Are the protocols appropriate and is the work technically sound?

Yes

Reviewer Expertise:

Cytogenetics and genomics of mosquitoes.

I confirm that I have read this submission and believe that I have an appropriate level of expertise to confirm that it is of an acceptable scientific standard, however I have significant reservations, as outlined above.

# Reviewer report: Reviewer response for version 1

**DOI:** 10.21956/wellcomeopenres.22690.r74126

**Published:** 2024-02-27

**Authors:** Chenyang Cai

**Affiliations:** 1 Chinese Academy of Sciences, Nanjing, China

The manuscript presents a comprehensive genome assembly of an individual male *Diloba caeruleocephala*, a member of the Noctuidae family. The assembly spans 762.5 megabases and is scaffolded into 31 chromosomal pseudo-molecules, including the Z sex chromosome. Additionally, the mitochondrial genome, spanning 15.36 kilobases, has been assembled.

 As usual, the manuscript presents a valuable resource in the form of a high-quality genome assembly of *Diloba caeruleocephala*, shedding light on its chromosomal organization and mitochondrial genome. With clear data visualization and comprehensive assembly statistics, the manuscript is well-positioned to contribute to the field of Lepidoptera genomics. I recommend acceptance of the manuscript as it is.

Are sufficient details of methods and materials provided to allow replication by others?

Yes

Is the rationale for creating the dataset(s) clearly described?

Yes

Are the datasets clearly presented in a useable and accessible format?

Yes

Are the protocols appropriate and is the work technically sound?

Yes

Reviewer Expertise:

phylogenomics, genomics

I confirm that I have read this submission and believe that I have an appropriate level of expertise to confirm that it is of an acceptable scientific standard.
